# Complete Genome Sequence of the Gamma-Aminobutyric Acid-Producing Strain *Streptococcus thermophilus* APC151

**DOI:** 10.1128/genomeA.00205-17

**Published:** 2017-04-27

**Authors:** Daniel M. Linares, Silvia Arboleya, R. Paul Ross, Catherine Stanton

**Affiliations:** a Food Biosciences Department, Teagasc Food Research Centre, Moorepark, Fermoy, County Cork, Ireland; b APC, Microbiome Institute, University College Cork, Cork, Ireland; c School of Microbiology, University College Cork, Cork, Ireland

## Abstract

Here is presented the whole-genome sequence of *Streptococcus thermophilus* APC151, isolated from a marine fish. This bacterium produces gamma-aminobutyric acid (GABA) in high yields and is biotechnologically suitable to produce naturally GABA-enriched biofunctional yogurt. Its complete genome comprises 2,097 genes and 1,839,134 nucleotides, with an average G+C content of 39.1%.

## GENOME ANNOUNCEMENT

*Streptococcus thermophilus* is a nonpathogenic homofermentative facultative anaerobic lactic acid bacterium with a long history of use in the artisanal and modern industrial manufacture of fermented dairy products, especially yogurt (1). In addition, this commensal bacterium is one of the pioneer colonizers of oral and small intestine mucosal surfaces in newborns (2) and remains predominant in the oropharyngeal (2, 3) and gastrointestinal (4) tracts throughout the human life span. Based on the potential beneficial effects on human health, it is sometimes marketed as a probiotic (5–9).

We here report the genome of *Streptococcus thermophilus* APC151, a strain isolated from the digestive tract of a marine fish that produces large amounts of gamma-aminobutyric acid (GABA) (10). GABA has been classified as a health-promoting bioactive component in foods and pharmaceuticals (11) due to its function as an antihypertensive and antidiabetic neurotransmitter (11–15).

Genomic DNA was purified using the GenElute bacterial genomic DNA kit (Sigma-Aldrich). The nucleotide sequence of *S. thermophilus* was resolved utilizing PacBio single-molecule real-time (SMRT) technology on an RS system (GATC Biotech, Konstanz, Germany). Whole-genome sequencing yielded a total of 72,472 reads with a mean length of 12,628 bp. Subsequent *de novo* assembly utilizing the HGAP3 protocol yielded a single polished contig with 423-fold average reference coverage.

Annotation was performed by the RAST server (16). The total genome size was 1,839,134 bp, which included 2,097 predicted open reading frames (ORFs) (2,012 coding genes and 85 RNA genes) and a G+C content of 39.1%. The genome encompasses one predicted phage packaging cassette, one predicted prophage lysogenic conversion module, three clustered regularly interspaced short palindromic repeat (CRISPR) clusters, and 12 genes related to transposases. Additionally, the genes responsible for GABA biosynthesis, encoding the glutamate decarboxylase (*gadB*) and the glutamate/GABA antiporter (*gadC*), have been identified in the genome of this strain. Sequence analysis revealed that the *gadB*-*gadC* genes are flanked by transposases, a genetic organization similar to other GAD^+^ genomes available in GenBank.

The strain was found to be sensitive to all antibiotics proposed by European Food Safety Authority (EFSA) standards (10). In addition, no amino acid decarboxylases associated with biogenic amines biosynthesis or virulence-related genes were identified in this genome.

The *S. thermophilus* APC151 genome showed a high degree of identity to that of strain *S. thermophilus* ND03 (GenBank accession no. NC_017563). The comparison of the two genomes revealed that strain APC151 contains an additional cluster of 13 genes (encoding a 5S RNA, a small subunit rRNA, a large subunit rRNA, and 10 tRNA genes) which are not present in the ND03 strain. In addition, strain APC151 acquired a number of mutations that disrupted or caused a frameshift in seven genes (encoding two transposases, two acyltransferases, the phosphate-transport-ATP-binding protein PstB, the β-carotene 15-15′-monooxygenase, and the substrate-specific component MtsA methionine-regulated ECF transporter).

These data are intended to increase the availability of the genomes of GABA-producing *S. thermophilus* strains in order to better understand their biodiversity and their potential technological, probiotic, and biofunctional properties.

### Accession number(s).

This whole-genome sequence was deposited at DDBJ/EMBL/GenBank under the accession number CP019935.
